# A20 Promotes Ripoptosome Formation and TNF-Induced Apoptosis via cIAPs Regulation and NIK Stabilization in Keratinocytes

**DOI:** 10.3390/cells9020351

**Published:** 2020-02-03

**Authors:** Maria Feoktistova, Roman Makarov, Sihem Brenji, Anne T. Schneider, Guido J. Hooiveld, Tom Luedde, Martin Leverkus, Amir S. Yazdi, Diana Panayotova-Dimitrova

**Affiliations:** 1 Department of Dermatology and Allergology, University Hospital RWTH Aachen, Pauwelsstraße 30, 52074 Aachen, Germany; mfeoktistova@ukaachen.de (M.F.); rmakarov@ukaachen.de (R.M.); sbrenji@ukaachen.de (S.B.); ayazdi@ukaachen.de (A.S.Y.); 2 Department of Medicine III, Department of Gastroenterology, Hepatology and Hepatobiliary Oncology, University Hospital RWTH Aachen, Pauwelsstraße 30, 52074 Aachen, Germany; anschneider@ukaachen.de (A.T.S.); tluedde@ukaachen.de (T.L.); 3 Nutrition, Metabolism & Genomics Group, Division of Human Nutrition & Health, Wageningen University, 6700 AA Wageningen; The Netherlands; guido.hooiveld@wur.nl

**Keywords:** cell death, keratinocytes, A20, NF-κB signaling, ripoptosome

## Abstract

The ubiquitin-editing protein A20 (TNFAIP3) is a known key player in the regulation of immune responses in many organs. Genome-wide associated studies (GWASs) have linked A20 with a number of inflammatory and autoimmune disorders, including psoriasis. Here, we identified a previously unrecognized role of A20 as a pro-apoptotic factor in TNF-induced cell death in keratinocytes. This function of A20 is mediated via the NF-κB-dependent alteration of cIAP1/2 expression. The changes in cIAP1/2 protein levels promote NIK stabilization and subsequent activation of noncanonical NF-κB signaling. Upregulation of TRAF1 expression triggered by the noncanonical NF-κB signaling further enhances the NIK stabilization in an autocrine manner. Finally, stabilized NIK promotes the formation of the ripoptosome and the execution of cell death. Thus, our data demonstrate that A20 controls the execution of TNF-induced cell death on multiple levels in keratinocytes. This signaling mechanism might have important implications for the development of new therapeutic strategies for the treatment of A20-associated skin diseases.

## 1. Introduction

Tumor necrosis factor (TNF) is a central cytokine with pleiotropic functions involved in the regulation of homeostasis, controlling inflammatory cytokine production, cell survival, and cell death [1]. Most of the biological effects of TNF are consequences of its binding to TNF receptor 1 (TNFR1). Signaling molecules such as TRADD, TRAF2, RIPK1, cIAP1, and cIAP2 are recruited to receptor complex I within minutes of the stimulation of TNFR1 [2,3,4]. The modification of the downstream signaling proteins by the ubiquitin system is an important switch point that directs the TNF signal towards either NF-κB activation and cell survival or cell death [5,6,7]. Depending on the molecular conditions in the cell, the formation of different types of complex II, namely IIa, IIb (the ripoptosome), or IIc (the necrosome) [8] follows the signaling from complex I [9]. The ripoptosome, which is formed upon the depletion of cellular inhibitors of apoptosis (cIAPs) and various extracellular stimuli [10,11], is another important switch point that commits the cell to either apoptosis or necroptosis.

A20 (also known as tumor necrosis factor-α-induced protein 3 (TNFAIP3)) was first identified in human endothelial cells as an important anti-inflammatory and anti-apoptotic molecule [12,13,14]. A20 is a zinc finger protein [15,16], the expression of which is strongly dependent on TNF-induced NF-κB activation. A20 possesses both ubiquitin ligase and deubiqitinase activities [17]. A20 removes K63 ubiquitin chains from RIPK1 in TNF-receptor complex I, thereby limiting TNF-induced NF-κB activation. Furthermore, it ligates K48 ubiquitin chains to RIPK1, thereby targeting it for proteasomal degradation. Additional to its role as NF-κB signaling regulator, A20 has a function in cell death signaling. Despite the broadly accepted anti-apoptotic role of A20, in certain circumstances, the protein performs pro-apoptotic functions [18,19].

A number of single nucleotide polymorphisms (SNPs) in *TNFAIP3* (A20), mainly affecting A20 expression, have been identified and linked to a number of inflammatory and autoimmune pathologies including rheumatoid arthritis (RA), systemic lupus erythematosus (SLE), inflammatory bowel disease (IBD), and psoriasis [20,21]. Moreover, upregulation of A20 expression has been reported in several cancers, such as inflammatory breast cancer, glioma, nasopharyngeal carcinoma, and squamous cell carcinoma [22,23,24,25].

In this study, we characterized the role of A20 in the regulation of TNF-induced cell death signaling in keratinocytes. We showed that an elevated level of A20 results in TNF-induced cell death, which is mediated by ripoptosome formation. In this setting, A20 plays a critical role in the regulation of both canonical and noncanonical NF-κB signaling. Our results suggest that canonical NF-κB activation and its target genes *BIRC2/3* (cIAP1/2) and *TRAF1* (TRAF1), but not *CFLAR* (cFLIP), are important checkpoints in A20-dependent TNF-induced cell death in keratinocytes. Our study thus provides significant insight into the critical role A20 plays in cell death regulation.

## 2. Materials and Methods

The following antibodies (Abs) and reagents were used for WB analysis: Abs for A20/TNFAIP3 (Novus Biologicals, Centennial, CO, USA) and caspase-8 (C-15; kindly provided by P.H. Krammer; C-20, Santa Cruz, Dallas, TX, USA); caspase-10 (MBL, Woburn, MA, USA); active caspase-3 (R&D, Minneapolis, MN, USA); caspase 3 (BD Bioscience, San Jose, CA, USA); cFLIP (NF-6; Alexis, San Diego, CA, USA); FADD, TRADD and RIP1 (Transduction Laboratories, San Diego, CA, USA); rat Abs against cIAP1 [26], cIAP2 [27], β-actin and β-tubulin (clone 2.1, Sigma, St. Louis, MO, USA); TRAF2 (Abcam, Cambridge, UK); IκBα and TNFR1 (Santa Cruz Dallas, TX, USA); pIκBα, p-p65, p100/p52, IKK2, and NIK (Cell Signaling, Danvers, MA, USA). Horseradish peroxidase (HRP)-conjugated goat anti-rabbit, goat anti-rat IgG, goat anti-mouse IgG Abs, and HRP-conjugated goat anti-mouse IgG1, IgG2a, IgG2b Abs were obtained from Southern Biotechnology Associates (Southern Biotechnology Associates, Birmingham, AL, USA). Necrostatin-1 was purchased from Sigma (Sigma, St. Louis, MO, USA). An IAP antagonist (compound A) was kindly provided by TetraLogics Pharmaceuticals (TetraLogics Pharmaceuticals, Phoenixville, PA,, USA). The pancaspase inhibitor Z-Val-Ala-DL-Asp-fluoromethylketone (zVAD-fmk) was purchased from Bachem GmbH (Bachem GmbH, Bubendorf, BL, Switzerland).

To express Fc-TNF, we used a previously published construct [28] which was provided by P. Schneider (University of Lausanne, Epalinges, Switzerland). HF-TNF was produced and purified as previously described [3].

### 2.1. Cell Culture

The spontaneously transformed HaCaT keratinocyte line was provided by Dr Petra Boukamp (DKFZ, Heidelberg, Germany). Cell lines were cultured as previously described [29]. HeLa cells were provided by Dr Michael Boutros (DKFZ, Heidelberg, Germany) and were cultured in DMEM containing 10% fetal calf serum (FCS).

### 2.2. Generation of Cell Lines

For retroviral (RV) and LV overexpression, the corresponding cDNAs were cloned into the pCFG5-IEGZ retroviral vector or PF 5x UAS MCS W SV40 Prom vector, respectively, by standard cloning procedures and verified by sequencing. Cells were selected for 10–14 days by zeocin selection or for 4 days by puromycin selection. The ectopic expression of the respective molecules was confirmed by FACS analysis and WB. Cells from two to six passages were used for subsequent analyses. Primary murine keratinocytes were isolated from the skin of newborn wild cFLIP^fl/fl^ mice and spontaneously immortalized in CnT-07 medium (CELLnTEC, Bern, Switzerland).

### 2.3. CRISPR Cell Line Generation

A20-KO cells were generated using the pSpCas9(BB)-2A-GFP (PX458) plasmid (Addgene, Town of Watertown, MA, USA). gRNA insertion was performed as described previously [30]. gRNA sequences targeting the 5′ end of the gene were designed using the open access software provided at http://crispr.mit.edu/. The gRNA sequences used were as follows:

Ah1: TTCCAGTGTGTATCGGTGCA

Ah2: AACCATGCACCGATACACAC

Two days post-transfection, the cells were sorted with a BD FACSAria I (BD Biosciences), and single clones were isolated and analyzed to confirm successful A20 KO.

### 2.4. Cell Stimulation Conditions

The following stimulation conditions were used throughout the experiments: prestimulation with zVAD-fmk (10 mM), necrostatin-1 (50 mM), or IAP antagonist (100 nM) alone or in their respective combinations for 1 h. HF-TNF stimulation concentrations for the crystal violet assay, propidium iodide (PI) staining, and immunofluorescence microscopy were as follows: HeLa cells—125 ng/mL; HaCaT cells and immortalized murine keratinocytes—250 ng/mL. For caspase-8 complex IP, cells were stimulated with 1 mg/mL HF-TNF for 2 h. For ligand affinity precipitation, the cells were stimulated with TNF-Fc supernatant for 5 min.

### 2.5. Western Blot Analysis

Five micrograms of total cellular protein was separated by SDS-PAGE on 4–12% gradient gels (Invitrogen, Karlsruhe, Germany) and then transferred to nitrocellulose or PVDF membranes. The membranes were blocked and incubated with primary and appropriate secondary Abs, as described previously [29]. Bands were visualized using an ECL detection kit (Amersham, Freiburg, Germany).

### 2.6. Crystal Violet Assay

Crystal violet staining of attached live cells was performed 18–24 h after stimulation with the indicated concentrations of HF-TNF as described in Section 2.4; the experiments were performed in 96 well plates with 3 wells per condition, as previously described [29]. The optical density (OD) of the control cultures was normalized to 100% and used to compare the stimulated cells.

### 2.7. Propidium Iodide Staining

A total of 2 × 10^4^ cells were stimulated as described in Section 2.4 for 18 h, trypsinized, washed with PBS, and stained with PI (10 µg/mL) for 15 min in the dark. The cells were analyzed on a BD Accuri C6 flow cytometer (BD Bioscience).

### 2.8. Immunofluorescence Microscopy

To detect the nuclear morphology and integrity of the cell membrane, 5 × 10^4^ cells of each cell line were seeded per well in a 12 well plate. Following 24 h of incubation for adherence, the cells were stimulated for 24 h as indicated above. Subsequently, the cells were incubated with Hoechst 33,342 (5 μg/mL; Polysciences Europe, Eppelheim, Germany) and SYTOX^®^ Green (5 pM; Invitrogen™, Molecular Probes™, Eugene, OR, USA) for 15 min at 37 °C, and then immediately observed by phase-contrast or fluorescence microscopy using a Zeiss Axio Observer A1 (Carl Zeiss Microscopy, Jena, Germany). Digital images were processed in an identical manner using ZEN2 (blue edition; Carl Zeiss Microscopy, Jena, Germany).

### 2.9. Coimmunoprecipitation of Caspase-8-Bound Complexes

In total, 1 × 10^7^ cells were washed once with medium at 37 °C and pre-incubated for 1 h with 100 nM IAP antagonist at 37 °C. Next, the cells were treated with 1 µg/mL HF-TNF for 2 h. The stimulation was stopped by washing the cells four times with ice-cold PBS. The cells were lysed on ice in 2 mL of lysis buffer (30 mM Tris-HCl pH 7.5 at 21 °C, 120 mM NaCl, 10% glycerol, 1% Triton X-100, and complete protease inhibitor cocktail (Roche Molecular Diagnostics, Mannheim, Germany)) for 30 min. The lysates were spun down twice—once at 20,000× *g* for 5 min and once at 20,000× *g* for 30 min. A small fraction of the cleared lysate was used as input control. Next, 1 μg of anti-caspase-8 antibody (C-20, Santa Cruz) was added. The caspase-8-containing complexes were precipitated from the lysates by coincubation with 40 μL of protein G beads (Roche) for 16–24 h on an end-over-end rotator at 4 °C. The precipitates were washed four times with lysis buffer. The protein complexes were eluted from dried beads by the addition of standard reducing sample buffer and boiled at 95 °C. Next, the proteins were separated by SDS-PAGE on 4–12% NuPAGE gradient gels (Invitrogen) and detected by WB analysis.

### 2.10. TNF-Fc Ligand Affinity Precipitation

A total of 1 × 10^7^ cells were washed once with medium at 37 °C and pre-incubated for 1 h with 100 nM IAP antagonist at 37 °C. Next, the cells were treated with 5 mL of TNF-Fc supernatant for 5 min. The cells were lysed and centrifuged as described above for caspase-8 containing complexes. Subsequently, 40 μL of TNF-Fc was added to the unstimulated lysates to precipitate the unstimulated receptors. Finally, the complexes were precipitated and washed as described for the precipitation of caspase-8-bound complexes.

### 2.11. Affymetrix GeneChip Oligoarray Analysis

Murine and human keratinocytes were starved for 6 h in medium without FCS and then stimulated with HF-TNF (500 ng/mL). Total RNA was isolated from the cells using an RNeasy kit (Qiagen, Hilden, Germany). The total murine and human RNA (100 ng per sample) was labeled using a Whole-Transcript Sense Target Assay kit (Affymetrix, Santa Clara, CA, USA) and hybridized to whole-genome Affymetrix GeneChip Mouse Gene 2.1 ST and Human Gene 2.1 ST arrays, respectively. The quality control and data analysis pipeline have previously been described in detail [31]. Briefly, the normalized expression estimates of probe sets were computed by a robust multiarray analysis (RMA) algorithm [32], as implemented in the Bioconductor library affyPLM. The probe sets were redefined using current genome definitions available from the NCBI database, resulting in the profiling of 25,4282 (mouse) and 29,635 (human) unique genes (custom CDF version 23) [33]. The cell-death-related gene list was obtained from the MGI database [34] and contained probe sets that were up- or downregulated by more than 1.2-fold. Microarray data were submitted to the Gene Expression Omnibus (accession number GSE128249).

### 2.12. Statistics

All data are expressed as the mean ± SEM. A two-tailed Student′s *t*-test for two groups was used to assess the significance of differences.

## 3. Results

### 3.1. Elevated A20 Expression Sensitized Human and Murine Keratinocytes to TNF-Induced Cell Death

**Figure 1:**
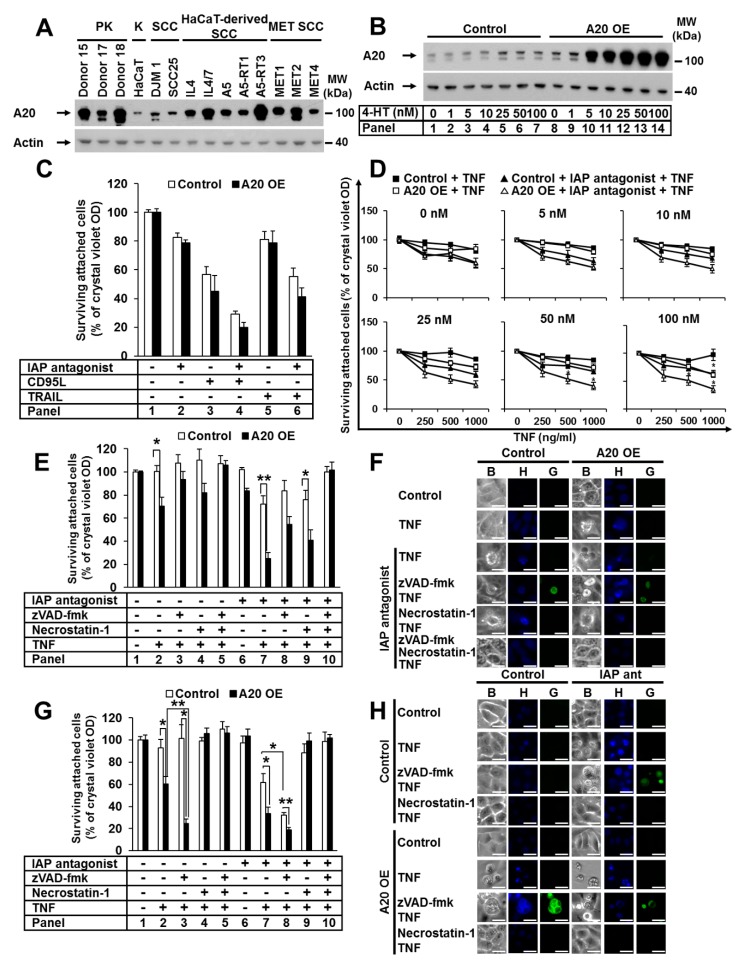
The elevated expression of A20 sensitizes human and murine keratinocytes to TNF-induced cell death. (**A**) Endogenous expression of A20 in PKs, HaCaTs (K), HaCaT-derived SCCs, DJM-1, SCC25, and cell lines from the intraindividual MET tumor progression model was analyzed by WB. (**B**–**E**) HaCaT cells were transduced with A20 LV, and A20 expression was induced with 4-HT. (**B**) A20 expression was induced with increasing 4-HT concentrations, and cell lysates were analyzed by WB. (**C**) Cells were stimulated as indicated, and their viability was analyzed by crystal violet assay. (**D**) A20 expression was induced with increasing 4-HT concentrations, and the cells were stimulated as indicated. Cell viability was analyzed by crystal violet assay. (**E**) Cells were stimulated as indicated, and cell viability was analyzed by crystal violet assay. (**F**) Cells were stimulated as indicated, and their death morphology was visualized by bright field “B” staining with Hoechst-33342 “H” and SYTOX Green “G”, immediately followed by transmission and fluorescence microscopy. The scale bars represent 100µm. (**G**,**H**) Immortalized murine keratinocytes transduced with RV stably expressing murine A20 were treated as described, and their cell viability and cell death morphology were analyzed by (**G**) crystal violet assay and (**H**) Hoechst-33342 and SYTOX Green staining as described for (**F**). The scale bars represent 100µm. For each diagram, the mean values (±SEM) of three independent experiments are shown. The WB and microscopy shown are representative of two independent experiments. In (**D**,**E**,**G**) * *p* < 0.05, ** *p* < 0.01.

A20 protects against TNF-induced cell death [35,36,37]. We investigated the expression of A20 in a set of primary and transformed human keratinocytes (PKs and HaCaTs) and squamous cell carcinoma (SCC) cells (Figure 1A). A heterogeneous expression profile of A20 was detected, with stronger expression in PKs than transformed keratinocytes. Additionally, we identified A20 expression upregulation in all analyzed SCC- and HaCaT-derived SCC cell lines compared to the parental HaCaTs, which was consistent with published data [25]. Next, we generated a HaCaT cell line with elevated A20 expression. To achieve a comparable level of A20 in HaCaT cells and SCC cell lines, we used a lentiviral (LV), 4-hydroxytamoxifen (4-HT)-inducible system (Figure 1B). Next, we examined the sensitivity of HaCaT cells with increased A20 levels to various death ligands, such as CD95L, TRAIL, and TNF. A20 overexpression did not change the sensitivity of HaCaT cells to CD95L and TRAIL (Figure 1C). Surprisingly, cells with increased amounts of A20 were significantly sensitized to TNF-induced cell death. The sensitization correlated with the amount of A20 in a dose-dependent manner (Figure 1D). Moreover, cell death was further significantly increased in the presence of an IAP antagonist (Figure 1D). Next, we analyzed the effect of the pancaspase inhibitor zVAD-fmk (zVAD) and the RIPK1 kinase inhibitor necrostatin-1 (Nec-1). Both inhibitors partially blocked cell death (Figure 1E, panels 8 and 9; Supplementary Figure S1A, panels 8 and 9), while the combination of both abolished it (Figure 1E, panel 10, Supplementary Figure S1A, panel 10). Western blot (WB) analysis confirmed the increased sensitization to cell death after TNF stimulation in the presence of IAP antagonist, demonstrated by enhanced caspase-8 and caspase-3 activation, as well as by RIPK1 and cFLIP cleavage (Supplementary Figure S1B, panels 4 and 8). Caspase-3 cleavage in response to TNF stimulation alone was detected (Supplementary Figure S1B, panel 6), suggesting apoptotic cell death. We next analyzed cell-death-associated morphology via fluorescent SYTOX Green/Hoechst staining. A20-overexpressing cells with typical apoptotic features, such as nuclear condensation and membrane blebbing with an intact cell membrane, were detected after TNF treatment (Figure 1F). In the presence of zVAD, the morphology converted from apoptotic to necroptotic, wherein cells exhibited a loss of membrane integrity, a lack of DNA condensation, and a rounded morphology (Figure 1F). The cells were largely rescued by treatment with the combination of zVAD and Nec-1 (Figure 1F). These data suggest that in A20-overexpressing cells with caspase inhibition, TNF stimulation results in RIPK1-dependent necroptosis.

To study the relevance of A20 in cell death sensitization in a different cell model, we immortalized primary murine keratinocytes and generated cell lines with elevated A20 expression (Supplementary Figure S1C). Consistent with our data obtained using human keratinocytes, the increase in A20 expression significantly sensitized murine keratinocytes to TNF-induced cell death, independent of the presence or absence of IAPs (Figure 1G, panels 2 and 7). In cells pretreated with zVAD, this sensitization was further increased (Figure 1G, panels 3 and 8). This particular effect of zVAD on cell death is specific to murine cells and has previously been reported for the L929 murine fibroblast cell line [38]. In both wild-type and A20-overexpressing murine keratinocytes, zVAD treatment further increased TNF-induced cell death, whereas Nec-1 treatment blocked it completely (Figure 1G). This finding suggests that TNF-induced apoptosis in the absence of IAPs critically requires the kinase function of RIPK1, as previously shown [9,38]. Consistently, keratinocytes with elevated A20 expression showed a typical apoptotic cell death morphology with condensed nuclei and cell blebbing upon TNF stimulation (Figure 1H). Wild-type cells showed the same morphology upon TNF stimulation in the presence of an IAP antagonist alone (Figure 1H). When caspases were blocked, the phenotype was converted to necroptosis (Figure 1H). Cell death was completely blocked upon pretreatment with Nec-1 (Figure 1H). Taken together, our data demonstrate that TNF-induced cell death in keratinocytes with elevated A20 expression is quantitatively and qualitatively equivalent to TNF-induced cell death in cells with depleted IAPs.

### 3.2. A20 Deficiency in Human Cells Did Not Change Their Sensitivity to TNF-Induced Cell Death

**Figure 2:**
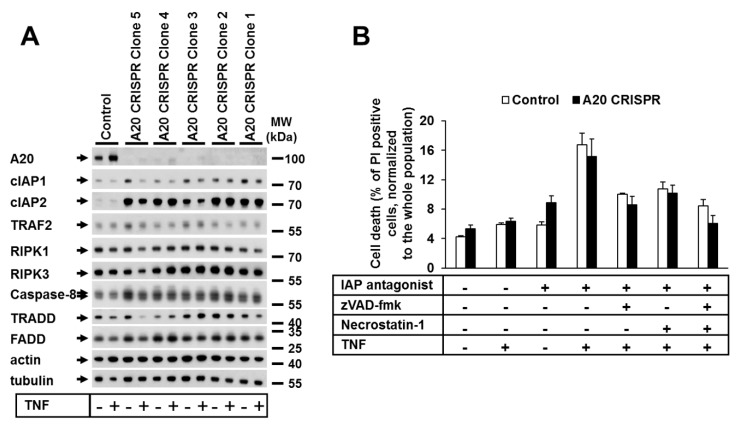
A20 deficiency in human cells did not change their sensitivity to TNF-induced cell death. (**A**) Protein expression in A20 CRISPR HaCaT clones analyzed by WB. (**B**) Selected clones from CRISPR HaCaT cells were treated as shown, and cell death was analyzed by PI staining and FACS analysis. Error bars represent the SEM of three independent experiments. The WBs shown are representative of two independent experiments.

Next, we investigated the impact of A20 deficiency on TNF-dependent cell death in human cells. We thus generated A20-deficient HaCaT and HeLa cell lines using CRISPR-Cas9 technology (Figure 2A; Supplementary Figure S2A). In contrast to the generally accepted role of A20 as an anti-apoptotic protein [39], in our study, the deletion of A20 in HeLa or HaCaT cells had no effect on the TNF-mediated cell death sensitivity (Figure 2B, Supplementary Figure S2B). The analysis of the protein expression patterns of both A20-deficient HaCaT and HeLa cells showed no alterations in the protein levels of cell death signaling-related molecules, with the exception of cIAP1 and cIAP2 (Figure 2A, Supplementary Figure S2A).

### 3.3. An Elevated Level of A20 Enhanced Ripoptosome Formation But Impairred TNF Complex I Formation

**Figure 3:**
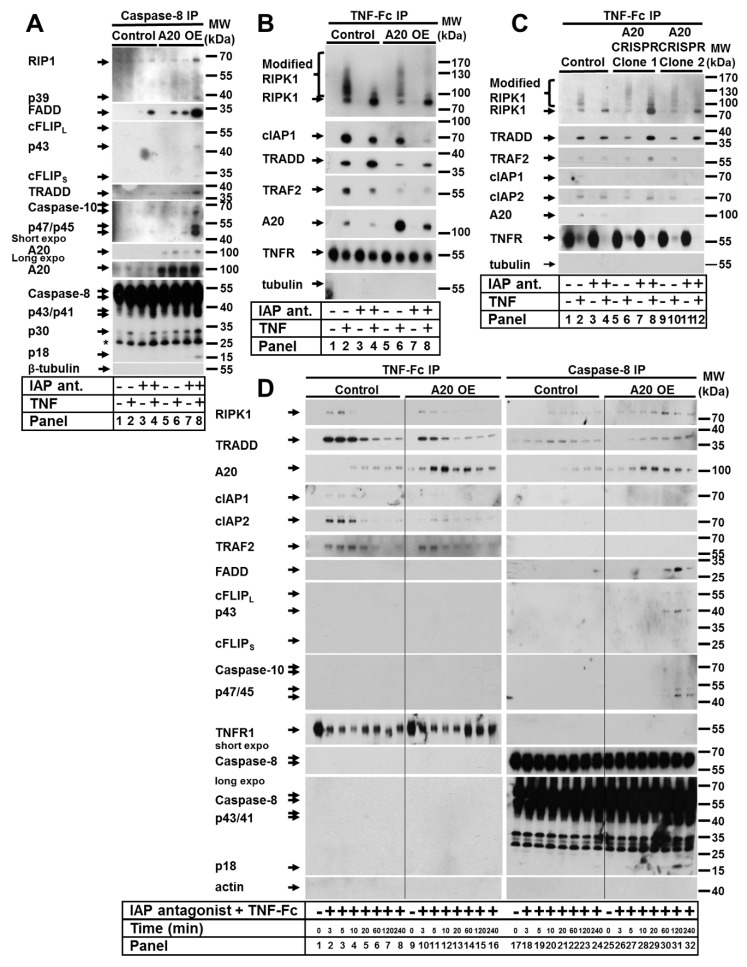
Elevated expression of A20 enhanced ripoptosome formation but impaired TNF complex I formation. (**A**,**B**,**D**) HaCaT cells were transduced with A20 LV. A20 expression was induced by 4-HT, and the cells were stimulated as indicated. (**A**) Caspase-8 immunoprecipitation (IP) and (**B**) TNF complex I precipitation were performed as described in the Materials and Methods. Recruited proteins were analyzed by WB. (**C**) Two A20 CRISPR HaCaT clones were treated as indicated, and TNF complex I precipitation was performed as described in the Materials and Methods. (**D**) TNF complex I and caspase-8 complexes were sequentially precipitated from the same cellular lysates and analyzed by WB.

Since the elevated expression of A20 appeared to control TNF-induced cell death in keratinocytes, we next aimed to dissect the molecular mechanisms responsible for the death signaling. Therefore, we immunoprecipitated the caspase-8 complex from cells stimulated with TNF in the presence or absence of an IAP antagonist (Figure 3A). We detected TNF-induced ripoptosome formation upon IAP depletion in control cells (Figure 3A, panel 4). Intriguingly, under the same conditions, the ripoptosome formation in cells with elevated A20 expression was greatly enhanced, as demonstrated by the increased interaction of caspase-8, FADD, TRADD, caspase-10, and RIPK1, as well as the detection of cFLIP in the complex (Figure 3A, panel 8). Importantly, TNF stimulation alone resulted in caspase-8–TRADD–FADD interactions (Figure 3A, panel 6). These data suggest that an elevated level of A20 represents a stimulus for ripoptosome formation upon TNF signaling. These findings were consistent with our cell death analysis results and suggest that TNF-induced cell death in A20-expressing cells is mediated by the ripoptosome.

Due to the negative regulatory role A20 plays in NF-κB signaling [17] and its recruitment to TNF complex I [40], we reasoned that an increase in A20 expression might also affect the formation of TNF complex I. Therefore, we next investigated the composition of TNF complex I in A20-overexpressing HaCaT keratinocytes in the presence and absence of an IAP antagonist (Figure 3B). TNF stimulation of control cells resulted in the recruitment of modified RIPK1 to complex I, whereas this modification was significantly reduced in the presence of the IAP antagonist (Figure 3B, panels 2 and 4). Additionally, cIAP1, TRADD, TRAF2, and A20 were recruited independently of IAP inhibition (Figure 3B, panels 2 and 4). Interestingly, in TNF-treated cells overexpressing A20, we detected decreased amounts of all recruited components, including less modified RIPK1, in the absence of the IAP antagonist (Figure 3B, panels 6 and 8). Thus, the elevated expression of A20 resulted in a reduction in TNF complex I formation. To determine whether a lack of A20 impacted TNF complex I formation, we immunoprecipitated the complex in A20-deficient HaCaT cells (Figure 3C). No significant difference in TNF complex I formation was observed between A20-deficient HaCaT cell clones and control cells. However, the RIPK1 ubiquitination pattern was changed in A20-deficient HaCaT cells, which was consistent with recent findings in A20-deficient mouse embryonic fibroblast (MEF) cells [39].

To further address the question of whether elevated levels of A20 suppress complex I formation or whether complex I is processed to complex II (the ripoptosome) at an increased rate, we performed a kinetic analysis of TNF complex I and complex II formation upon cIAP1/2 depletion. In cells with elevated A20 expression, the recruitment of TNF complex I components was reduced at all analyzed time points (Figure 3D, left panel). However, when we analyzed the ripoptosome formation in cells with elevated A20, we found that the recruitment of the ripoptosome components was increased and started much earlier than that in wild-type cells. All analyzed components were detected 60 min after TNF and IAP antagonist stimulation, in contrast to the control, where all analyzed components were detected at 240 min (Figure 3D, right panel). Together, these data demonstrate that A20 overexpression does not result in the increased speed of complex I components (TRADD and RIPK1) transition to complex II, but rather, as we previously suggested [10], the components are recruited to the ripoptosome independently and may not originate from TNF complex I.

### 3.4. A20-Mediated TNF-Induced Cell Death in Keratinocytes Is Dependent on Canonical NF-κB Signaling and cIAP1/2

**Figure 4:**
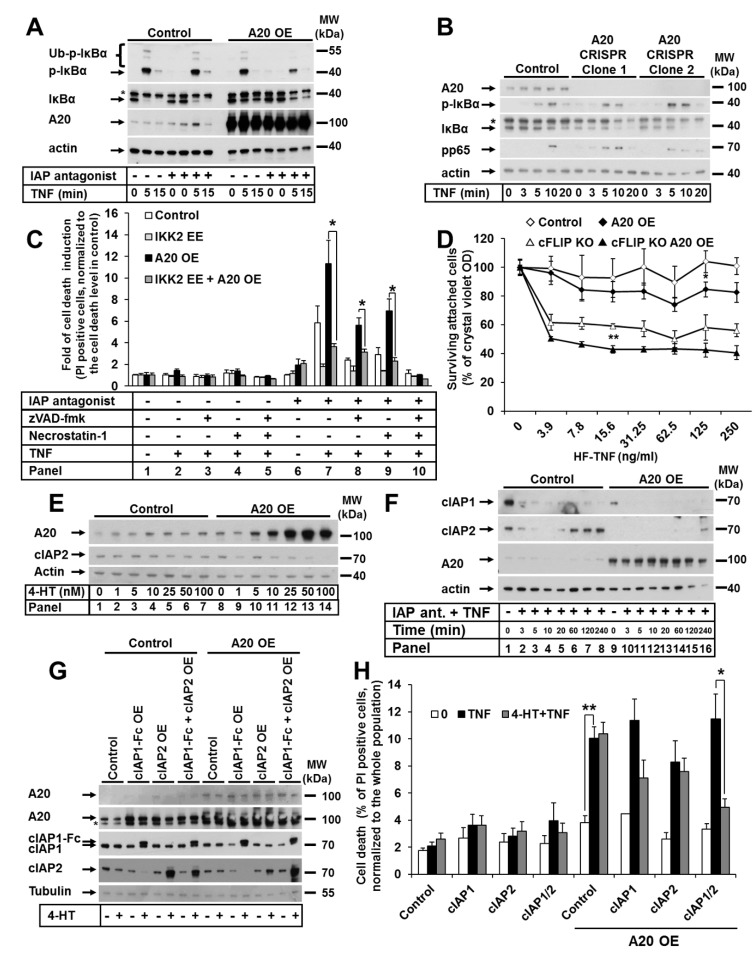
A20-mediated TNF-induced cell death in keratinocytes is dependent on canonical NF-κB signaling and cIAP1/2, but not cFLIP. (**A**) HaCaT cells were transduced with A20 LV, and A20 expression was induced by 4-HT. The cells were stimulated as indicated, and cell lysates were analyzed by WB. (**B**) A20 CRISPR HaCaT cells were treated as indicated, and the cell lysates were analyzed by WB. (**C**) HaCaT cells were sequentially transduced with LV containing IKK2 EE (inducible expression), and RV containing A20 (stable expression). The cells were treated as indicated, and cell death was analyzed by PI staining and FACS. (**D**) Immortalized murine cFLIP KO keratinocytes were transduced with LV containing murine A20. Cells were treated as indicated, and cell survival was analyzed by crystal violet assay. (**E**) HaCaT cells were transduced with A20 LV, and A20 expression was induced by treatment with increasing 4-HT concentrations. cIAP2 expression was analyzed by WB. (**F**) Cells from (**E**) were treated as indicated and analyzed by WB. (**G**) HaCaT cells were sequentially transduced with A20 RV (stable expression) and LV containing cIAP1-Fc or cIAP2, or a combination of both (inducible expression). Cell lysates were analyzed by WB. (**H**) Cell lines from (**G**) were treated as indicated, and cell death was analyzed by PI staining and FACS. In each diagram, the mean values of three independent experiments (±SEM) are shown. In (**C**,**D**,**H**) * *p* < 0.05, ** *p* < 0.01. The WBs are representative of two independent experiments.

Since the most well-characterized function of A20 is its regulation of NF-κB signaling, we next examined the effect of A20 overexpression on the activation of canonical NF-κB signaling in HaCaTs. As expected, upon TNF stimulation, cells with elevated A20 expression had downregulated canonical NF-κB signaling, which was validated via the decreased IκBα phosphorylation and degradation at all analyzed time points (Figure 4A), independent of the presence of IAP antagonist. Consistently, we observed increased NF-κB activation in A20-deficient keratinocytes, as demonstrated by the rapid phosphorylation and degradation of IκBα and more rapid and prolonged p65 phosphorylation (Figure 4B). Since the sensitivity of cells with elevated A20 expression to TNF-induced cell death was increased and canonical NF-κB activation was impaired, we hypothesized that this sensitization resulted from the blockade of NF-κB signaling. Therefore, we generated a HaCaT cell line with increased A20 expression and the 4-HT-inducible expression of a constitutively active IKK2 mutant (IKK2 EE), leading to constant hyperactivation of NF-κB signaling (Supplementary Figure S3A). Indeed, cells with the 4-HT-induced expression of the IKK2 EE mutant were protected against TNF-induced cell death under IAP depletion conditions, independent of A20 expression (Figure 4C). Consistent with these results, the overexpression of an IKK2 kinase-dead mutant (IKK2 KD) that blocks NF-κB signaling caused no further sensitization to TNF-induced cell death in A20-overexpressing cells (Supplementary Figure S3B,C). The reduction in NF-κB activation in cells with elevated A20 levels was confirmed by the impaired upregulation of canonical NF-κB target genes expression upon TNF stimulation in both human and murine keratinocytes (Supplementary Figure S3D). In summary, these data indicate that A20 mediates TNF-induced cell death through the regulation of the canonical NF-κB pathway, with the subsequent expression of NF-κB target genes.

Since cFLIP is a well-known NF-κB target gene that regulates caspase-dependent cell death [41,42,43], we next investigated its role in the regulation of cell death in keratinocytes with increased A20 expression. To this end, A20 was overexpressed in immortalized primary keratinocytes isolated from cFLIP^fl/fl^ animals. The lentiviral transduction of Cre-expressing constructs caused the deletion of the cFLIP locus (Supplementary Figure S3E). As expected, cFLIP deletion caused a significant increase in TNF-induced cell death (Figure 4D). Importantly, cell death was further increased by the upregulation of A20 expression (Figure 4D). These data suggest that the mechanism of A20-mediated, TNF-induced cell death is independent of cFLIP.

We observed that upon upregulation of A20 expression in keratinocytes, the expression level of cIAP2 was reduced in a dose-dependent manner (Figure 4E). Since *BIRC3* (cIAP2) is an NF-κB target gene, the amount of cIAP2 protein was diminished in cells treated with the IAP antagonist and was restored after 1 h of TNF treatment (Figure 4F). Interestingly, this response was not observed in cells with elevated A20 levels (Figure 4F). Moreover, A20-knockout (KO) cells displayed increased amounts of cIAP1 and cIAP2 (Figure 2A). We thus reasoned that cIAPs might contribute to cell death regulation in cells with increased levels of A20; therefore, we established cell lines with the inducible expression of both cIAP1-Fc and/or cIAP2 (Figure 4G). Cell death analyses revealed that the expression of cIAP1 partially protected cells with elevated A20 expression from TNF-induced cell death (Figure 4H). The protection of A20-overexpressing cells that expressed cIAP2 was reduced (Figure 4H). The combined expression of both cIAPs significantly protected against cell death. This may be explained by the compensatory expression of cIAP1 and cIAP2 in HaCaT cells (Supplementary Figure S3F), as we have previously described [29]. Taken together, these data suggest that cIAP1/2 levels in A20-overexpressing cells are critical effectors of TNF-induced cell death.

### 3.5. The Noncanonical NF-κB Pathway Was Activated in Keratinocytes with Elevated A20 Expression

**Figure 5:**
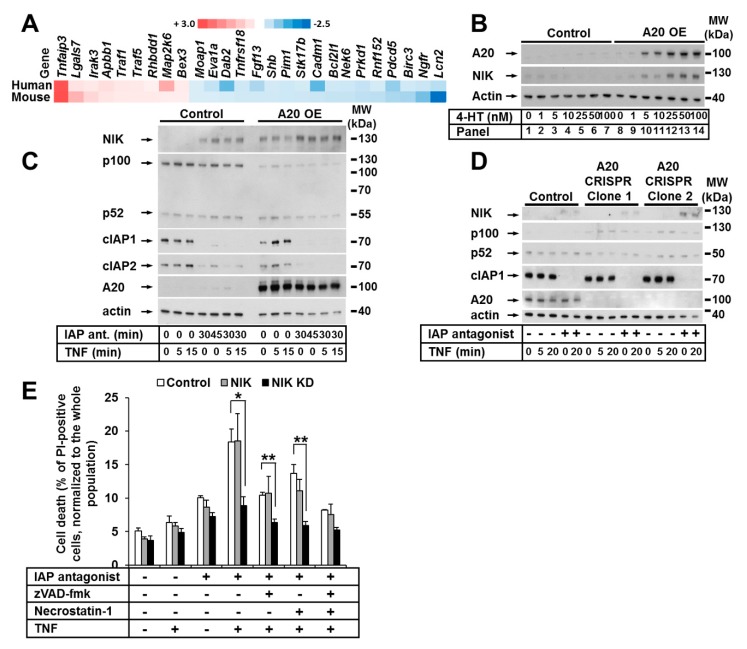
The noncanonical NF-κB pathway was activated in keratinocytes with elevated A20 expression. (**A**) Heat maps of apoptosis-related genes with altered expression in A20-overexpressing human (n = 2) and mouse (n = 2) keratinocyte cells compared to their relative control cells. (**B**) HaCaT cells were transduced with A20 LV. A20 expression was induced by treatment with increasing 4-HT concentrations. Cell lysates were analyzed by WB. (**C**) HaCaT cells were transduced with A20 LV. A20 expression was induced with 4-HT. The cells were stimulated as indicated, and NIK expression was analyzed by WB. (**D**) A20 CRISPR HaCaT cells were treated as indicated, and cell lysates were analyzed by WB. The WBs are representative of two independent experiments. (**E**) HaCaT cells were transduced with RV containing NIK or NIK KD. The cells were stimulated as shown, and cell death was analyzed by PI staining and FACS analysis. Mean values (±SEM) of three independent experiments are shown in (**E**) * *p* < 0.05, ** *p* < 0.01.

To investigate the impact of A20 on gene expression in murine and human keratinocytes independent of species specificity, we performed cross-species gene expression profile comparisons. Focusing on cell death, we detected approximately 26 transcripts that were similarly dysregulated in both mouse and human keratinocytes (Figure 5A). Interestingly, we observed the significant upregulation of TNF-stimulation-induced TNF-associated factor-1 (TRAF1) expression in both murine and human keratinocytes (Figure 5A). TRAF1, which is similar to TRAF2 and TRAF3, is involved in the regulation of NIK stabilization and the regulation of noncanonical NF-κB signaling [44,45,46]. Indeed, in cells with an increased A20 level, a simultaneous dose-dependent accumulation of NIK (without any other stimuli) was detected (Figure 5B). Thus, we next explored the effect of A20 on noncanonical NF-κB signaling in HaCaT keratinocytes. To this end, we treated HaCaT keratinocytes with TNF and the IAP antagonist and confirmed the previously found [47] accumulation of NIK in the presence of the IAP antagonist, indicative of noncanonical NF-κB activation (Figure 5C). As expected, NIK accumulation was further enhanced by IAP depletion in A20-overexpressing cells compared to control cells (Figure 5C). Importantly, in cells with elevated A20 expression, the ratio of nonprocessed p100 to its active form p52 was shifted towards p52 (Figure 5C) [47,48]. Since p100 processing and NIK accumulation are markers of noncanonical NF-κB activation [49,50], our results suggest that the overexpression of A20 may initiate noncanonical NF-κB signaling. Consistent with these data, we observed decreased activation of the noncanonical NF-κB pathway in A20-deficient cells, as demonstrated by the reduced p100 processing and consequent accumulation (Figure 5D). It was recently reported that NIK contributes to TNF-dependent ripoptosome formation and cell death [51]. To explore whether NIK kinase activity is essential for cell death execution in the absence of IAPs in keratinocytes, we next generated HaCaT cell lines expressing a kinase-dead mutant of NIK (NIK KD). In contrast to the cells expressing functional NIK, the NIK KD HaCaT cells were significantly protected from TNF-induced cell death in the presence of the IAP antagonist (Figure 5E).

**Figure 6:**
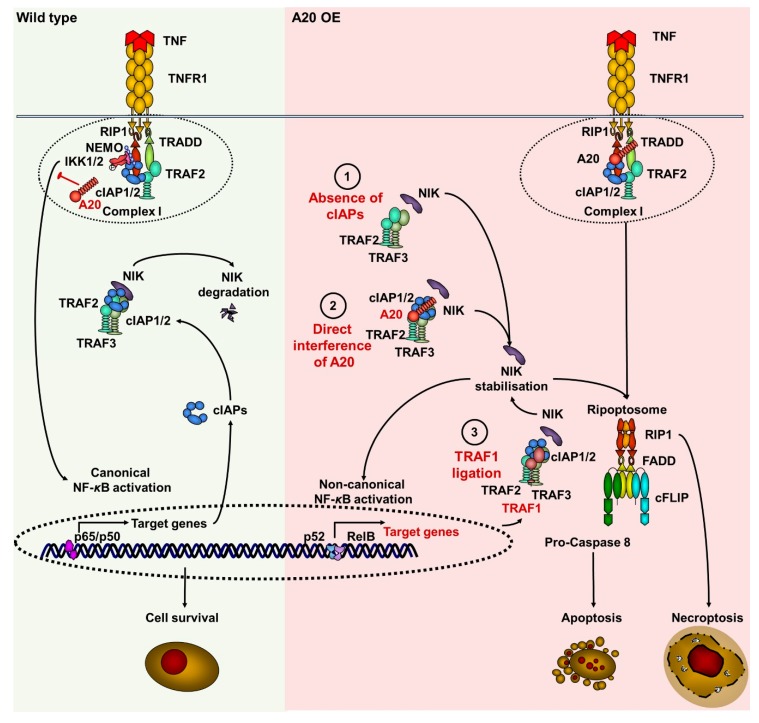
Suggested model of A20-mediated TNF-induced cell death signaling via the regulation of cIAPs, TRAF1, and NIK in keratinocytes. In wild-type conditions (green field), upon stimulation of TNF receptor, the TNF receptor complex I is formed by recruitment of TRADD, TRAF2, cIAPs, and RIPK1. RIPK1 is ubiquitinated within complex I, followed by further recruitment of the NEMO/IKK1/2 complex. This leads to the activation of canonical NF-kB signaling, followed by the expression of target genes, including (*BIRCs*) cIAPs. cIAP1/2, TRAF2 and TRAF3 form a complex with NIK, targeting it to degradation. A20 blocks the activation of canonical NF-kB through deubiquitination of RIPK1. The elevated amounts of A20 (pink field) can stabilize NIK via the following scenarios: (1) A20 blocks TNF-induced canonical NF-κB activation, thereby repressing the expression of target genes, including (*BIRCs*) cIAPs. Similarly to the effect of IAP antagonist, the A20-dependent depletion of cIAPs blocks TRAF2/TRAF3 complex, and prevents the degradation of NIK, causing its stabilization. (2) A20 directly interacts with cIAP1, allowing for NIK dissociation from the TRAF2/TRAF3 complex. This results in NIK stabilization, which promotes the noncanonical NF-κB activation and the expression of its target gene (*TRAF1*) TRAF1 in an autocrine manner. (3) Upregulated TRAF1 interacts directly with the TRAF2/TRAF3 complex and stabilizes NIK by preventing its degradation. Finally, the kinase activity of NIK promotes ripoptosome formation. The ripoptosome can direct the cell to either apoptosis or necroptosis.

In summary, increased A20 expression resulted in NIK stabilization and the activation of noncanonical NF-κB signaling. These data indicate that A20 plays a critical role in the regulation of both canonical and noncanonical NF-κB signaling in keratinocytes, and support our hypothesis that A20 can regulate TNF-induced cell death at several molecular levels (Figure 6).

## 4. Discussion

Proper cell and tissue development and homeostasis are maintained through the tight regulation of cell proliferation and cell death. Many different cellular platforms control signaling upstream of NF-κB activation (for example, the TNF-R1 complex I, the NOD complex, and the FADDosome), or cell death (for example, TNF complexes II; TRAIL-R and CD95 DISC, and the ripoptosome) and appear to be critically important for this regulation [52,53]. As A20 is known to be recruited to several cellular signaling complexes in both the inflammatory and cell death pathways, it is conceivable that it plays a key role in the regulation of cellular homeostasis. Although A20 is involved in the negative regulation of canonical NF-κB signaling [54], its role in the control of cell death appears controversial. Numerous reports have described A20 as an anti-apoptotic molecule [13,14,39]; however, some studies have proposed that A20 can act as a pro-apoptotic factor [18,19,55]. Here, we studied the role of A20 in death receptor (DR)-induced cell death signaling in keratinocytes. We found that elevated A20 expression significantly increased the sensitization of cells to TNF-induced cell death, but did not alter sensitivity to TRAIL or CD95-L. These differences in sensitivity to various cell death ligands might be explained by the most prominent function of TNF being the induction of inflammatory signaling through NF-κB activation, whereas the primary role of TRAIL and CD95L is apoptosis induction. A20 overexpression sensitized both human and murine keratinocytes to apoptosis. When apoptosis was blocked, cell death was converted from apoptosis to necroptosis. This cell death phenotype strongly resembles the outcome of DR or TLR3 stimulation in cIAPs-depleted conditions, mediated by ripoptosome formation [10,29]. A20-mediated cell death sensitivity was further enhanced by the depletion of cIAP1/2 in both human and murine keratinocytes. Therefore, we suggest that increased levels of A20 may represent a further signal for ripoptosome formation that results in cell death in keratinocytes. This hypothesis is supported by our data demonstrating TNF-induced ripoptosome formation in HaCaT keratinocytes overexpressing A20, which was significantly increased in the absence of cIAP1/2. Furthermore, we found that the increased expression of A20 blocked the restoration of cIAP2 protein levels during IAP antagonist treatment, whereas the cIAP2 expression level was normally restored in approximately 60 min (Figure 4F [29]). This effect was probably due to the inhibition of canonical NF-κB signaling. Finally, our biochemical and mRNA analyses revealed changes in the expression level of cIAP2 as a direct consequence of A20 expression level modification. This suggests that A20 is a natural regulator of cIAPs expression, which might be of critical relevance for cell death execution. This notion is supported by our data showing the protective properties of cIAP1 and, to a lesser extent, cIAP2 in cells with an elevated A20 protein level. Since the best protection was achieved by the overexpression of both cIAPs, based on present and previous observations [29], we speculate that the compensatory regulation of cIAP1 and cIAP2 expression might be involved. The significance of cIAP1 and particularly its UBA domain for TNF-induced cell death was recently shown [7]. In this report, cIAP1 was shown to play a critical role in RIPK1 phosphorylation and polymerization as well as the formation of cell death signaling complexes, such as the ripoptosome or necrosome [7,56,57].

In cells with impaired activation of the canonical NF-κB pathway, the expression of its target gene (*CFLAR*) cFLIP was also compromised, resulting in increased cell death [2]. Importantly, we showed here that in cells with elevated A20 expression and impaired NF-κB activation, cFLIP was not a factor in TNF-induced cell death.

Epithelial cells constantly interact with danger signals, which might cause their particular sensitivity to changes in cellular A20 levels. A similar sensitivity to TNF-induced cell death has also been demonstrated in intestinal epithelial cells (IECs) overexpressing A20. However, the studies in IECs failed to detect a correlation between the A20-mediated regulation of NF-κB signaling and cell death [55]. Significantly, we showed that in keratinocytes, the inhibition of canonical NF-κB signaling in cells overexpressing A20 directly resulted in increased cell death, which was blocked by the restoration of NF-κB signaling. Furthermore, A20 was also found to be important for the induction of noncanonical NF-κB signaling. Elevated A20 levels were correlated with NIK stabilization in keratinocytes, which may have occurred as a direct consequence of the reduced cIAPs levels. We showed here that the degradation of cIAPs promoted NIK stabilization and NIK-kinase-activity-mediated cell death. This eventually results in the activation of noncanonical NF-κB signaling [47,58]. Additionally, the direct interaction of A20 with cIAP1 is thought to impact the dissociation of the TRAF2/TRAF3 complex, which also results in NIK stabilization and the activation of noncanonical NF-κB signaling [59]. Significantly, our data suggest the contribution of an additional mechanism involving the A20-dependent upregulation of TRAF1 expression. TRAF1 is an important factor for the dissociation of the TRAF2/TRAF3 complex and NIK stabilization [46]. Consistent with our findings, a recent study demonstrated that NIK plays an unexpected but significant role in the control of TNF-induced RIPK1/caspase-8 complex formation upon IAP depletion and cell death [51]. Here, we identified NIK kinase activity as being critical for the control of TNF-mediated cell death in keratinocytes.

The general biological role of A20 in keratinocytes is the regulation of canonical and noncanonical NF-κB signaling. TNF-induced cell death or cell survival of human and murine keratinocytes depends on the interplay between these two pathways. Our data on A20-deficient keratinocytes demonstrated no difference in the cell death sensitivity. However, A20-deficient cells showed upregulation of the canonical NF-κB signaling (Figure 4B). Since the canonical NF-κB pathway activated pro-survival and inflammatory response in keratinocytes and HeLa cells, A20 KO cell lines did not show a sensitization to TNF stimulation. Based on our A20 overexpression studies in keratinocytes, a protection from TNF-induced cell death would be expected upon deletion of A20. However, since both HaCaT and HeLa cells are fully insensitive to TNF treatment, no further protection was observed in A20 KO cells. Despite the fact that IAP antagonist sensitized both HaCaT and HeLa cells to TNF-induced cell death, no difference in the sensitivity was observed in A20 KO cells. This finding might be explained by our hypothesis that the cIAPs function in the NIK-stabilization signaling cascade is downstream of A20 (Figure 6).

In summary, we describe that A20, despite its reported anti-apoptotic properties, can sensitize keratinocytes to TNF-induced cell death via multilevel regulation, resulting in ripoptosome formation. Our data provide insights into the recognition and understanding of the switch points that control cell fate and determine cell survival or cell death, which might be essential for the further development of treatment strategies for TNF-associated skin pathologies.

## Supplementary Materials

The following are available online at https://www.mdpi.com/2073-4409/9/2/351/s1, Figure S1: An increase in A20 expression results in reduced viability of HaCaT cells in response to TNF, in the presence and absence of IAP antagonist: Figure S2: Loss of A20 in HeLa cells does not alter cell death sensitivity. Figure S3: TNF-induced cell death in keratinocytes with elevated A20 expression is linked with the regulation of canonical NF-κB signaling.
