# Supplementary material for: A20 Promotes Ripoptosome Formation and TNF-Induced Apoptosis via cIAPs Regulation and NIK Stabilization in Keratinocytes
Source: Cells. 2020 Feb 3;9(2):351. doi: 10.3390/cells9020351 (PMC7072579; doi:10.3390/cells9020351)
Supplement: Supplementary file 1 [file cells-09-00351-s001.pdf]

SUPPORTING INFORMATION

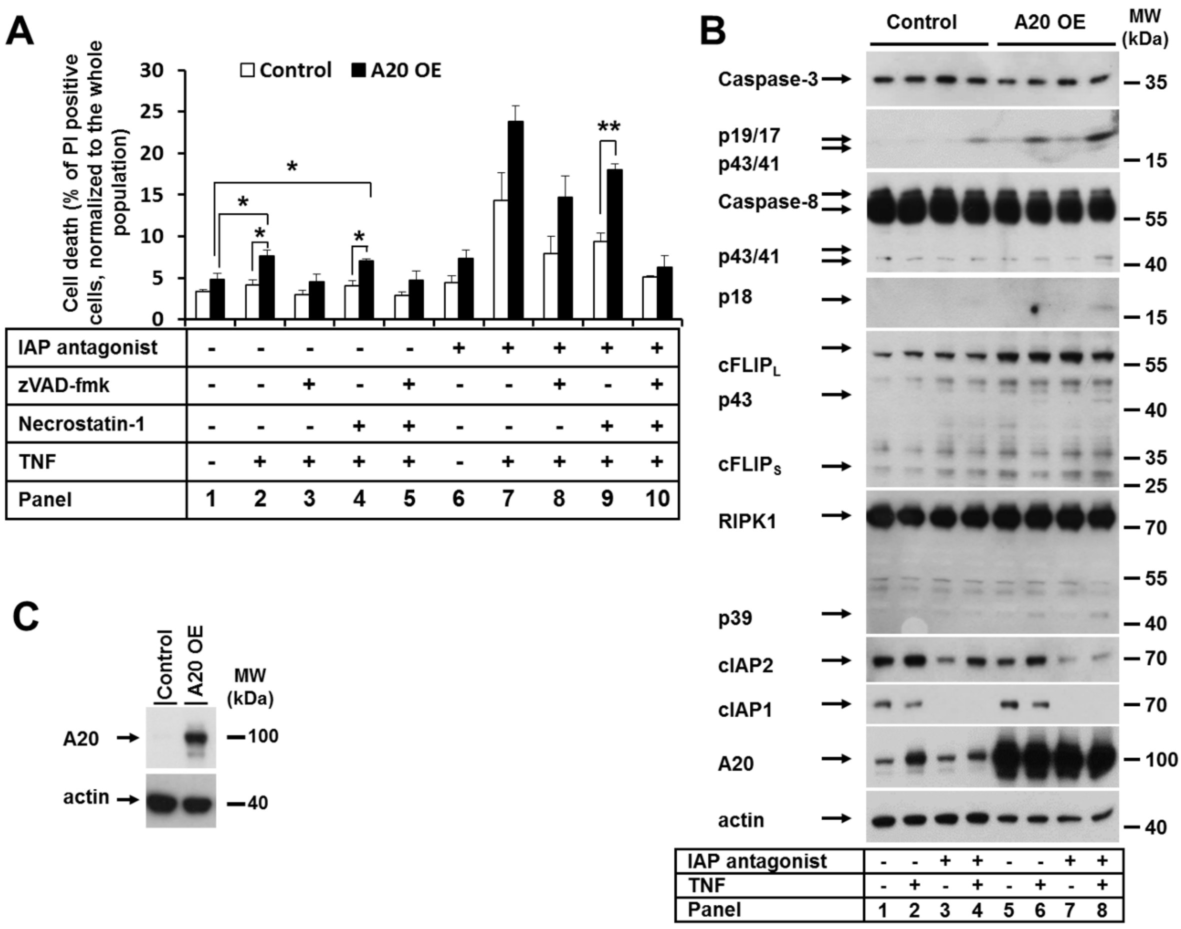

Fig. S1. An increase in A20 expression results in reduced viability of HaCaT cells in response to TNF, in the presence and absence of IAP antagonist. (**A, B**) HaCaT cells were transduced with A20 LV and A20 expression was induced by 4-HT. (**A**) The cells were stimulated as indicated and cell death analyzed by PI staining and FACS analysis. Average values of three independent experiments (±SEMs) are shown, * p<0,05, **p<0,01. (**B**) A20 transduced HaCaT cells were treated as indicated and proteins of interest were analyzed by WB. (**C**) Murine keratinocytes were transduced with RV vector containing murine A20 and cellular lysates were analyzed by WB.

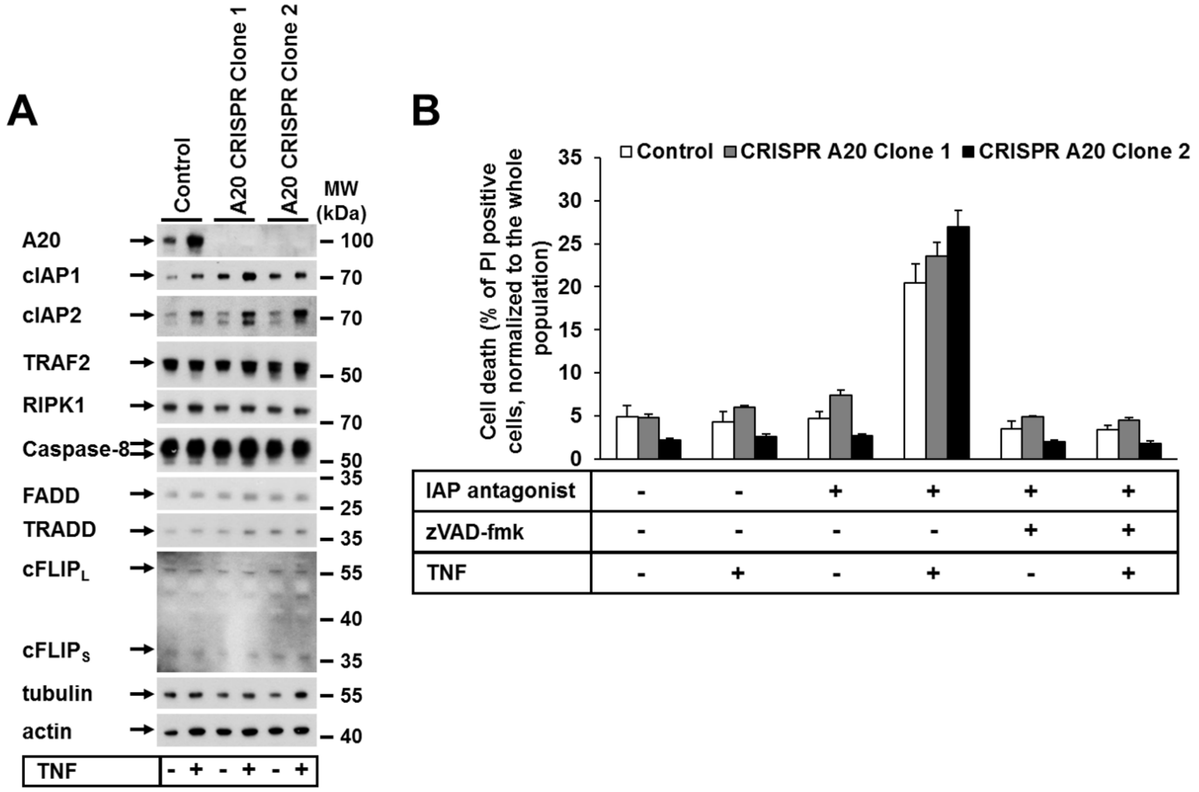

IAP antagonist	-	-	+	+	+	+
zVAD-fmk	-	-	-	-	+	+
TNF	-	+	-	+	-	+

Fig. S2. Loss of A20 in HeLa cells does not alter cell death sensitivity. (**A**) Protein expression in control and A20 CRISPR HeLa clones were analyzed by WB. (**B**) Selected A20 CRISPR HeLa clones were treated as shown and cell death was analyzed by PI staining and FACS analysis. Mean values (±SEMs) of 3 independent experiments are shown. The WBs shown are representative of 2 independent experiments.

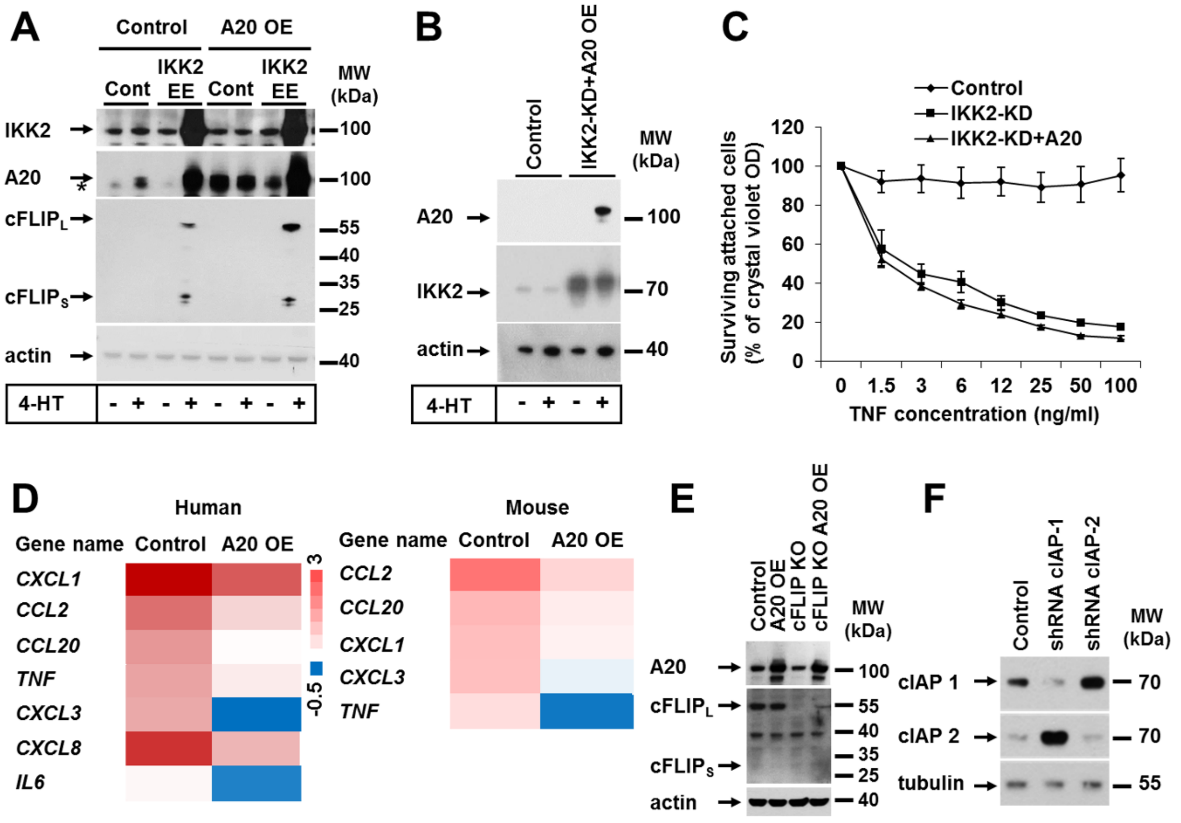

Fig. S3. TNF-induced cell death in keratinocytes with elevated A20 expression is linked with the regulation of canonical NF-κB signaling. (**A**) HaCaT cells were sequentially transduced with LV containing IKK2 EE (inducible expression) and RV containing A20 (stable expression). IKK2 EE expression was induced by 4-HT and the proteins of interest were analyzed by WB. (**B**) HaCaT cells were sequentially transduced with RV containing IKK2 KD (stable expression) and LV containing 4-HT- A20 (inducible expression). A20 expression was induced with 4-HT and cell lysates were analyzed by WB. (**C**). Cells from (B) were treated with the indicated concentrations of HF-TNF for 18 hours. Cell viability was analyzed by crystal violet assay. Values from 3 independent experiments (±SEMs) are shown. (**D**) Heat maps of NF-κB target genes altered upon TNF stimulation of human (n=2), and mouse (n=2) keratinocytes with elevated A20 expression. GEO accession number GSE128249. (**E**) Murine A20 or control vector were expressed in spontaneously immortalized keratinocytes isolated from cFLIP$^{fl/fl}$ animals. The cFLIP locus was deleted via LV expression of Cre recombinase. Cell lysates were analyzed by WB. (**F**) Compensatory regulation of cIAP1 and cIAP2 expression. HaCaT cells were transduced with LV containing shRNA for cIAP1 and cIAP2, and their expression status was investigated by WB.
